# Perinatal determinants of germ-cell testicular cancer in relation to histological subtypes

**DOI:** 10.1038/sj.bjc.6600470

**Published:** 2002-08-27

**Authors:** L Richiardi, O Akre, R Bellocco, A Ekbom

**Affiliations:** Department of Medical Epidemiology, Karolinska Institute, Berzelius väg 15C, SE-17177 Stockholm, Sweden; Unit of Cancer Epidemiology and Center for Oncologic Prevention, University of Turin, V. Santena 7, 10126 Torino, Italy; Unit of Clinical Epidemiology, Department of Medicine, Karolinska Hospital, L1:00 SE-171 76 Stockholm, Sweden; Department of Epidemiology, Harvard School of Public Health, Boston, USA

**Keywords:** testicular cancer, seminoma, non-seminoma, perinatal exposures, maternal hormones, aetiological heterogeneity

## Abstract

We aimed to investigate the role of perinatal determinants on the risk for germ-cell testicular cancer, with respect to the aetiological heterogeneity between seminomas and non-seminomas. A case–control study of 628 case patients with testicular cancer (308 seminomas and 320 non-seminomas) and 2309 individually matched controls was nested within a cohort of boys born from 1920 to 1980 in two Swedish regions (Uppsala-Örebro Health Care Region and Stockholm). Cases were diagnosed from 1958 to 1998 and were identified through the Swedish National Cancer Registry. Perinatal information on cases and controls was collected through charts available at maternity wards. Gestational duration, categorised in three categories (<37, 37–41, >41 weeks), was negatively associated with the risk for testicular cancer (*P* value for linear trend=0.008). A protective effect of long gestational duration and an increased risk for high birth weight were found for seminomas. Non-seminomas were associated with short gestational duration, particularly among those with low birth order (odds ratio: 3.02, 95% confidence intervals: 1.53–5.97) and high maternal age (odds ratio: 2.33, 95% confidence intervals: 1.19–4.55). No significant differences were found in tests for heterogeneity between the two histological groups. Our data support the hypothesis that intrauterine environment affects the risk for germ-cell testicular cancer. Seminomas and non-seminomas seemed to have similar risk patterns, although they are not entirely congruent.

*British Journal of Cancer* (2002) **87**, 545–550. doi:10.1038/sj.bjc.6600470 www.bjcancer.com

© 2002 Cancer Research UK

## 

Testicular cancer is the most common neoplasm among young adults, with a peak of occurrence among males aged 30 years. The incidence varies 10-fold between countries (Adami *et al*, 1994) and has been increasing markedly in several populations in the last 50 years (Schottenfeld, 1996; Moller, 2001; Power *et al*, 2001). The trend is a birth cohort phenomenon rather than a calendar period or age effect (Bergstrom *et al*, 1996; Davies, 1981; Liu *et al*, 2000).

Undescended testis is the only established risk factor apart from heredity and genetic disorders, and the pattern of occurrence of testicular cancer indicates that prenatal life is a critical window for susceptibility to carcinogenic exposures (Henderson *et al*, 1979; Sharpe and Skakkebaek, 1993; Ekbom, 1998). A number of studies have investigated the role of different indicators of prenatal and perinatal exposures, referring to the hypothesis that the risk for germ-cell testicular cancer is associated with the levels of sex hormones during gestation (Schottenfeld *et al*, 1980; Depue *et al*, 1983; Brown *et al*, 1986; Moss *et al*, 1986; Swerdlow *et al*, 1987a; Akre *et al*, 1996; Moller *et al*, 1996; Moller and Skakkebaek, 1997; Petridou *et al*, 1997; Sabroe and Olsen, 1998; Wanderas *et al*, 1998; Weir *et al*, 2000; Dieckmann *et al*, 2001). Birth order, duration of gestation, birth weight, nausea and bleeding during pregnancy, neonatal jaundice, placental weight and maternal age have been reported as risk factors for testicular cancer by at least one paper, but there is a marked inconsistency.

Most of the studies on testicular cancer have treated the neoplasm as a single entity, while few of them conducted analyses separated by the two major histological groups: seminoma and non-seminoma (Moss *et al*, 1986; Swerdlow *et al*, 1987a; Akre *et al*, 1996; Moller *et al*, 1996; Moller and Skakkebaek, 1997; Wanderas *et al*, 1998; Weir *et al*, 2000). In general, results on aetiological heterogeneity between the two types of testicular cancer have not been consistent.

In this study we have combined data from the Swedish Cancer Registry with information from maternity ward charts to perform a nested case–control study of perinatal factors in relation to risk for testicular cancer. The study is an enlargement of a previous investigation (Akre *et al*, 1996), and the aim was to increase statistical power in order to enable separate studies of testicular seminomas and non-seminomas.

## MATERIALS AND METHODS

### Study population

We conducted a case–control study nested in a cohort of males born in Sweden from 1920 to 1980 and still alive and resident in the country at 1 January 1958, when the National Cancer Registry was established. Subjects included in the cohort were delivered at hospitals in Uppsala-Örebro Health Care Region as well as in the city of Stockholm. The cohort is estimated to include the majority of all males delivered among residents in the catchments area of the hospitals in 1920–1940 and close to 100% hereafter, as home deliveries were rare events after 1940.

Cases of malignant germ-cell testicular cancer (code 178 in the International Classification of Diseases, 7th Revision) were identified through the National Cancer Registry, that includes all newly diagnosed malignant neoplasms in Sweden from January 1958. They were diagnosed from 1958 to 1996, with the exception of subjects born in Uppsala-Örebro Health Care Region, for whom the period of follow-up ended in 1994.

Patients are listed in the Cancer Registry according to the national registration number (NRN) that was assigned to all residents in Sweden since 1947. NRN is a unique personal identifier containing information on date and county of birth for subjects born 1947 onwards, or county of residence at 1947 for those born before that (Lunde *et al*, 1980).

The NRN allowed us to select testicular cancer patients with the code for any of the six counties of Uppsala-Örebro Health Care Region as well as for the city of Stockholm. Underascertainment of cases could only occur for individuals who move out of their county of birth before 1947 or emigrated before being diagnosed with testicular cancer. Among potentially eligible subjects we selected those born in one of the hospitals that defined the cohort.

We identified 670 cases through the Cancer Registry. Among them, nine twins were excluded because twin pregnancies are associated with an altered foetal environment and, due to small numbers, it was not possible to adjust for twin status in the analysis. Seventeen subjects aged less then 15 years or more than 54 years at the time of diagnosis were also excluded as testicular cancer occurring before puberty or at old age can involve different etiological factors and pathologic mechanisms.

Information from the National Cancer Registry allowed us to assign cases to one of the two major groups of testicular cancer: seminoma and non-seminoma. The latter included cancers of mixed histological pattern. Tumours with a histopathologic code indicating origin other than germ cells were excluded from the study. Altogether, 628 cases remained for analysis.

Controls were the first four singleton male offspring born at the same hospital after a case subject. A linkage, based on the NRN, between our cohort and the Swedish Cancer Registry as well as the Swedish Death Registry was performed to verify that controls were alive and without testicular cancer at the time of diagnosis of the corresponding case. These criteria were met by 2309 controls.

The study was approved by the local ethics committee at Karolinska Institutet (n. 00-033).

### Exposure information

We collected information available at different maternity wards and their archives. A standardised chart to record information about newborns and their parents was introduced in Sweden in 1973. Hospitals included in the study were selected because they used similar charts before 1973, but the number of available variables varied over different hospitals and different time periods. However, the maternity charts were usually carefully filled in and missing values rarely occurred once a variable was included in the chart.

Variables used in the analysis included maternal age at delivery, socio-economic status of the mother according to educational and/or occupational level, maternal parity (defined as number of births including the present birth), pre-eclampsia or eclampsia during pregnancy, gestational duration and weight at birth, neonatal jaundice and medical problems of the newborn. Other recorded variables were not used either because of a large number of missing values (e.g. placental weight) or because they were not judged to be biologically plausible risk indicators.

Small- and large-for-gestational-age were computed on the basis of both weight at birth and gestational duration, according to the intrauterine growth curves reported by Marsál *et al* (1996). The values corresponding to the mean weight for gestational duration ±2 standard deviations were chosen as a cut-off to define respectively large- and small-for-gestational-age.

### Statistical methods

We estimated odds ratios (ORs) with corresponding 95% confidence interval (CI) using conditional logistic regression (Breslow and Day, 1980) available in SAS procedure PHREG (Sas Institute Inc, 1997). All multivariable models, adjusted for age and place of birth, included maternal age, socio-economic status (three categories: ‘high’, denoting college education; ‘medium’, white-collar worker or farm owner without college education; ‘low’, blue-collar workers), birth order, gestational duration and dimension-for-gestational-age. Birth weight was introduced in the models as alternative to dimension-for-gestational-age to avoid over-adjustment. Variables were treated as categorical as shown in Table 1

**Table 1:**
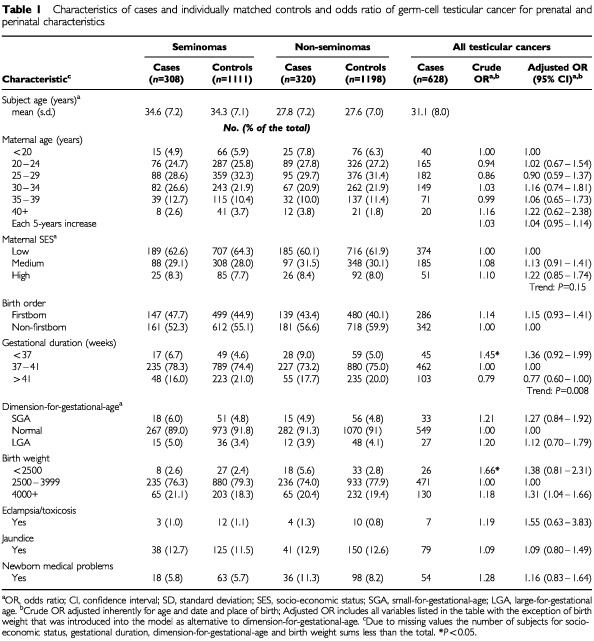
Characteristics of cases and individually matched controls and odds ratio of germ-cell testicular cancer for prenatal and perinatal characteristics

. Subjects with missing information in one or more variables were excluded from the analyses. If the subject was a case we also excluded the corresponding set of controls (Breslow and Day, 1980). A missing-indicator category was used instead for 18 cases (2.9%) and 53 controls (2.3%) with missing socio-economic values (Huberman and Langholz, 1999).

Most of the analyses were conducted separately for seminomas (308 cases, 1111 matched controls) and non-seminomas (320 cases, 1198 matched controls). Heterogeneity of results between the two histological types was tested by comparing the likelihood of the model with interaction term between histological group and determinant of exposure with the same parameter derived from the model without interaction term (likelihood ratio test). Variables were included in the models if they were independent determinants and/or substantial confounders.

The effect of individual and joint exposure indicators (maternal age, birth order and gestational duration) was also assessed. In this analysis maternal age was categorised into two groups according to the median value of the control distribution: young mothers (<28 years old) and old mothers (28+ years old).

## RESULTS

Characteristics of cases and individually matched controls are shown in Table 1. As expected seminoma cases had a higher mean age at diagnosis compared with non-seminomas. Table 1 also presents crude and adjusted odds ratios (ORs) for testicular cancer as a single entity. We found a statistically significant negative association with gestational duration (*P* for linear trend: 0.008), as well as a significant elevated risk for high birth weight.

In Table 2

**Table 2:**
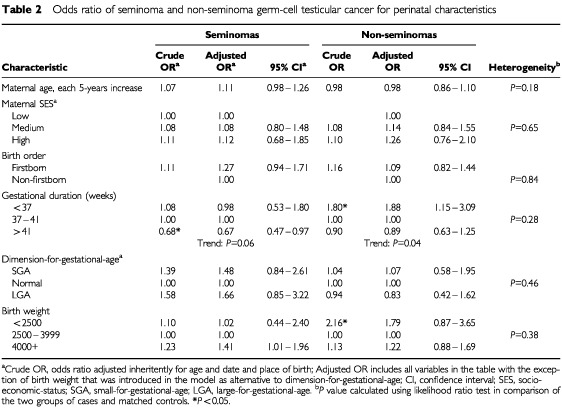
Odds ratio of seminoma and non-seminoma germ-cell testicular cancer for perinatal characteristics

results are presented stratified by the two histological types. There were no statistically significant differences between the two groups (see last column of Table 2). Although gestational duration showed a negative trend for both histological groups, we found that short duration of gestation was significantly associated with non-seminomas (OR: 1.88, 95% CI: 1.15–3.09), whereas a protective effect of long gestational duration was confined to seminomas (OR: 0.67, 95% CI: 0.47–0.97). A significant positive association was found between high birth weight and seminoma cases. Furthermore, we found a non-significantly increased risk for non-seminomas among boys with low birth weight.

As foetal growth and gestational duration are highly interdependent, we evaluated the joint effect of these variables in a separate model (data not shown in tables). As compared with those born normal-for-gestational-age at term, infants born both preterm (before gestational week 37th) and large-for-gestational-age had a three-fold risk for testicular seminomas (OR of 3.29, 95% CI: 1.23–8.80; based on eight exposed cases).

Table 3

**Table 3:**
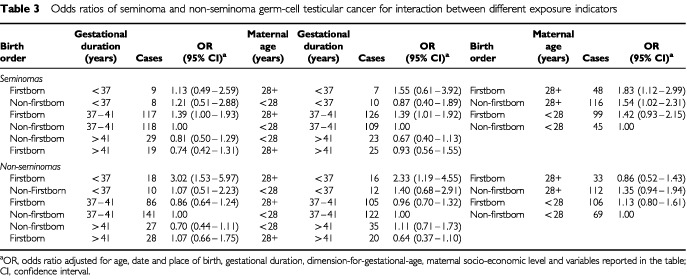
Odds ratios of seminoma and non-seminoma germ-cell testicular cancer for interaction between different exposure indicators

presents the individual and joint effects of the most relevant exposures included in the study. Among infants born at term, we found significantly elevated risks for seminomas among firstborns (OR: 1.39, 95% CI: 1.00–1.93; column 4) and boys with older mothers (OR: 1.39, 95% CI: 1.01–1.92; column 8). High maternal age was associated with an increased risk for seminomas both among firstborn and non-firstborn infants and the effect of maternal age and birth order appeared to be additive (column 12).

Among boys born preterm, high maternal age as well as low birth order further increased the risk for non-seminomas (OR of 2.33, 95% CI: 1.19–4.55 and OR of 3.02, 95% CI: 1.53–5.97 for high maternal age and low birth order respectively; see columns 8 and 4).

## DISCUSSION

### Contribution, strength and limitation

The present study is one of the largest to investigate the effect of different indicators of intrauterine exposures on the risk for germ-cell testicular cancer.

The study was an enlargement of a previous analysis that indicated etiological heterogeneity between the two major histological groups of testicular cancer (Akre *et al*, 1996). In the previous study the risk for seminomas was associated with high birth and placental weight, while an increased risk for non-seminomas was found for high maternal socio-economic status, short gestational duration, neonatal jaundice and low birth weight. Power limitations, however, hampered conclusive inference on heterogeneity. We therefore expanded the geographic base of the study and collected information on new cases and controls to triple the study size and increase the power. For instance, our present study has a power of 80% to detect an interaction ratio between the ORs estimated in the two histological groups of at least 1.7, with a significant level of 0.05, for a dicothomic variable, such as birth order. With the same power, the previous study (232 cases and 904 controls) achieved to estimate a significant interaction ratio of 2.4. Power calculation on the other variables produced similar conclusions.

Cases were identified through registries whose completeness was close to 100% and controls were selected from the study base in a randomized fashion.

A misclassification in the histological information recorded in the Cancer Registry between seminomas and non-seminomas may have occurred to some extent, resulting in a dilution of our estimates of the etiological differences between the two histological groups. Some types of non-seminomas, such as mixed tumours, may have been classified as seminomas, in particular up to 1976 when the work of the British Testicular Tumor Panel introduced a first standard in the classification system (Pugh, 1976). However, there are reasons to believe that this had only minor impacts on our results. Firstly, the difference of about 7 years between the mean age at diagnosis of the two types of testicular cancer is in line with results of other studies where the specimens were subjected to a pathological review (Weir *et al*, 2000). Furthermore, patients aged below 30 years at diagnosis, which are mostly non-seminomas, had a similar proportion of non-seminomas when diagnosed before 1976 (74.7% of 79 patients) and when diagnosed after that (73.2% of 213). Stratified analyses of those diagnosed after 1976 did not change our results more than marginally.

Exposure data were prospectively collected. The nested design of the present study permitted to preserve the validity of cohort studies. However, some variables had a high proportion of missing values because of the lack of standardised maternity charts before 1973. These indicators, such as placental weight, were excluded from the analysis.

Several tests were performed throughout the analyses, introducing a problem of multiple comparisons. In the present study, this issue is particularly relevant as there are not strong *a-priori* etiological hypotheses to separate the patients by the two groups, seminomas and non-seminomas. Thus, positive findings should be interpreted with caution, either in the frame of previous evidence or as a suggestion for further investigation.

### Testicular cancer as a single entity

Our data supported previous evidence that prenatal and perinatal exposures play a role in determining the risk for testicular cancer (Ekbom, 1998). The mechanism may be related to early failures in the process of differentiation of germ cells generating carcinomas *in situ* of the testis that can develop in testicular cancers during adult life (Dieckmann and Skakkebaek, 1999). The causal factors of testicular cancer are largely unknown. However it has been suggested that the risk may be increased by foetal exposure to both endogenous and exogenous oestrogens (Henderson *et al*, 1979; Sharpe and Skakkebaek, 1993). Several case–control studies, a few animal studies (Walker *et al*, 1990) and some investigations on foetal exposure to exogenous hormones (e.g. diethylstilbestrol (Strohsnitter *et al*, 2001)) lend indirect support for this hypothesis.

Results on gestational duration (negative association) and birth weight (u-shaped trend) from this study can be interpreted both in terms of oestrogen exposure and of foetal growth retardation or placental malfunction. Some previous studies found similar results for at least one of the two variables (Depue *et al*, 1983; Brown *et al*, 1986; Moller *et al*, 1996; Moller and Skakkebaek, 1997; Weir *et al*, 2000), but the inconsistency is marked. Since gestational duration and birth weight are strongly correlated, it is difficult to disentangle the effects of these two aspects of the intrauterine growth. However, we could estimate the risk for being small- and large-for-gestational-age because information on a large number of cases was available. As a result, it was found that the duration of gestation is a stronger predictor than birth weight.

One of the most frequently reported findings in previous studies is the increased risk of testicular cancer among firstborn infants (Depue *et al*, 1983; Swerdlow *et al*, 1987a; Prener *et al*, 1992; Moller and Skakkebaek, 1997; Sabroe and Olsen, 1998; Wanderas *et al*, 1998; Weir *et al*, 2000). We also found an increased risk, although not statistically significant.

In our previous study jaundice was found to be associated with an increased risk for testicular cancer, in particular for non-seminomas (Akre *et al*, 1996). Since then a Norwegian study has reported the same finding, using prospectively collected data (Wanderas *et al*, 1998). We could not confirm this association in the present study. However, the diagnosis of neonatal jaundice is based on symptomatic characteristics and the propensity to make the diagnosis have varied over different regions and periods. The high prevalence of jaundice among cases and controls in our study indicates low specificity and thus the lack of association may be due to a non-differential misclassification.

### Heterogeneity between seminomas and non-seminomas

The classification of germ-cell testicular cancer into seminoma and non-seminoma has a well established prognostic relevance. Despite the two histological types differing by about 10 years in the mean age at diagnosis, they show similar trends in incidence. Thus, it has been suggested that the aetiological heterogeneity is unlikely to be large (Moller, 1993), although a recent Canadian study found some differences between the two groups in the birth cohort pattern of the increase in incidence (Liu *et al*, 2000).

Some case–control studies conducted analysis separated by seminomas and non-seminomas (Moss *et al*, 1986; Swerdlow *et al*, 1987a,b; Prener *et al*, 1992; Akre *et al*, 1996; Moller and Skakkebaek, 1996, 1997; Sabroe and Olsen, 1998; Wanderas *et al*, 1998; Coupland *et al*, 1999; Weir *et al*, 2000), but results were not consistent and only part of them were in favour of etiological heterogeneities (Moss *et al*, 1986; Swerdlow *et al*, 1987a,b; Akre *et al*, 1996; Coupland *et al*, 1999). Most of these previous studies included a limited number of subjects with a little power to perform subgroup analyses.

In spite of the size of our enlarged study we could not find any statistically significant heterogeneity between seminomas and non-seminomas. However, tests for heterogeneity imply a multiplicative model and ruling out differences on the base of a formal test would be excessively conservative (Greenland and Rothman, 1998). Furthermore, the use of proxy variables such as birth order and foetal growth introduces distortion that may hide differences with respect to the ‘true’ exposures.

We investigated the effect of the interaction between different perinatal factors in analysis separated by the two histological groups. Seminomas were associated with high birth weight, in particular among preterm boys, and we found an additive effect between maternal age and birth order. These results are consistent with some previous studies that indicated at least one of the latter two variables as risk factors confined to seminomas (Swerdlow *et al*, 1987a; Prener *et al*, 1992; Moller and Skakkebaek, 1997; Sabroe and Olsen, 1998; Weir *et al*, 2000). Furthermore, both an English (Swerdlow *et al*, 1987a) and a Danish (Moller and Skakkebaek, 1997) study observed a high risk for testicular cancer among firstborn sons of older women. However, a recent study found an excess of seminomas and non-seminomas among firstborns of mothers aged less than 25 years, which is not consistent with our results (Weir *et al*, 2000).

Since variables that we examined in our study are only indicators of some unknown risk factors, possibly hormonal, any hypothesis on the underlying aetiological mechanism is tentative. Indicators for high levels of maternal oestrogens during pregnancy, such as high birth weight (Kaijser *et al*, 2000), being firstborn (Bernstein *et al*, 1986) and intermediate maternal age (Panagiotopoulou *et al*, 1990) have been associated with an increased risk for seminomas (Akre *et al*, 1996). Intrauterine growth retardation on the other hand has been seen as a proxy for low oestrogen levels and has been associated with an increased risk for non-seminomas (Akre *et al*, 1996). The association between premature birth and risk for non-seminomas that we found in our study is in line with this hypothesis. However, this study failed to put in evidence a significant difference between the two histological groups, suggesting that the etiological heterogeneity, if it exists, is not straightforward. The effect of short gestational duration on non-seminomas was positively modified by both low birth order and high maternal age. Hence, a more complex etiological mechanism related to the interaction between different perinatal exposures may have an effect on determining the type of germ-cell tumour.

### Conclusions

In conclusion, our results support the hypothesis that the intrauterine environment affects the risk for germ-cell testicular cancer. Seminomas and non-seminomas seemed to have similar risk patterns, although different patterns appear for the two histological groups with respect to interaction between different perinatal exposures.
